# Enhanced Photocatalytic Activity of CdS-Decorated TiO_2_/Carbon Core-Shell Microspheres Derived from Microcrystalline Cellulose

**DOI:** 10.3390/ma9040245

**Published:** 2016-03-29

**Authors:** Xin Liu, Yinliang Li, Jun Yang, Bo Wang, Mingguo Ma, Feng Xu, Runcang Sun, Xueming Zhang

**Affiliations:** 1 Beijing Key Laboratory of Lignocellulosic Chemistry, Beijing Forestry University, Beijing 100083, China; xin_liu@bjfu.edu.cn (X.L.); lylhappy@bjfu.edu.cn (Y.L.); yangjun11@bjfu.edu.cn (J.Y.); mzlwb@bjfu.edu.cn (B.W.); mg_ma@bjfu.edu.cn (M.M.); xfx315@bjfu.edu.cn (F.X.); rcsun3@bjfu.edu.cn (R.S.); 2 State Key Laboratory of Pulp and Paper Engineering, South China University of Technology, Guangzhou 510640, China

**Keywords:** functional composites, cellulose, photocatalysis, titanium dioxide, environmental degradation

## Abstract

The fabrication of reusable and biodegradation materials from renewable resources such as cellulose is essential for a sustainable world. The core-shell structured CdS-decorated TiO_2_/Carbon microspheres (CdS/TiO_2_/Carbon MS) photocatalyst was synthesized with controlled hydrolysis and a novel sonochemical method. It was prepared by using crosslinked microcrystalline cellulose as the core, tetrabutyl titanate as the titania source and CdS as the photosensitizer. The morphology, chemical structure and properties of the obtained material were characterized by many means. Additionally, the photocatalytic activity of the CdS/TiO_2_/Carbon MS was evaluated by the photodegradation efficiency of Rhodamine B solution, which reached 95.24% under visible light irradiation. This study demonstrated the excellent photocatalytic performance of CdS/TiO_2_/Carbon MS, which might have promising applications in environmental treatments.

## 1. Introduction

Water is the most widely distributed substance on our planet, and it plays a vital role in both human life and the environment [1]. During recent decades, the increasingly serious pollution of water resources as a global problem has received extensive attention [2]. Particularly, the wastewater containing persistent organic pollutants (POPs), such as various kinds of organic dyes, is not readily destroyed by conventional degradation methods [1,3]. As a consequence, it is imperative to develop environmentally friendly and efficient technologies for the degradation of these types of organic pollutants in wastewater [4].

Advanced oxidation processes (AOPs) have been regarded as an effective method for treating textile wastewater, which refers to a chemical treatment to oxidize and mineralize almost any organic contaminant [5]. Nevertheless, the process costs may be considered as the main obstacle to their commercial application. For avoiding this disadvantage, photocatalysis has emerged [6,7]. As a well-known semiconductor with a broad band gap (3.0 eV for rutile and 3.2 eV for anatase), titanium dioxide (TiO_2_) has been proven the most suitable candidate for widespread applications due to its fascinating properties such as low price, high chemical and thermal stability, non-toxicity, biocompatibility and excellent degradation capacity [8].

However, TiO_2_ has two crucial factors that limit its practical use. The first is that exciton creation can only be achieved with UV light. The second is the high recombination rate of the photogenerated electron-hole pairs [9,10]. Therefore, we need to pursue effective methods to improve the photocatalytic performance of TiO_2_ and utilize the visible light energy. Modification of TiO_2_ to enhance the photocatalytic activity is of interest and the surface deposition of metal or metal compound nano-particles (NPs) is widely adopted due to many factors: metal oxide particles on the TiO_2_ surface can support the charge separation and improve the formation of the free hydroxyl radicals [11,12,13]. Many metal sulfides (such as Bi_2_S_3_, CdS, MoS_2_ and WS_2_) and metal oxides (such as Bi_2_O_3_, Cu_2_O, Fe_2_O_3_, MoO_3_, SnO_2_, WO_3_, ZnO and ZrO_2_) have been reported to couple with TiO_2_ to form heterojunction photocatalysts with enhanced photocatalytic performance [14].

Besides, the surface structure of the photocatalyst is widely regarded as a key for selectivity and photocatalytic activity since most catalytic reactions are carried out when the reactants are adsorbed onto the catalyst surface [15]. In the last years, some work has been reported on the preparation of hybrid nanocomposites based on polymer matrix [16]. Using polymer as a substrate to immobilize the TiO_2_ can control the growth and nucleation of TiO_2_ particles [17,18]. The obtained materials offer unique properties over traditional materials on optical and mechanical properties because of the synergistic effects resulting from the chemical and physical interactions which occur between the inner layer and outer components [19]. Cellulose as the most abundant polymer on the earth has some promising properties, such as mechanical robustness, non-toxicity, hydrophilicity, biocompatibility and biodegradability [20]. Particularly, the microcrystalline cellulose (MCC) possesses unique properties typical of nanomaterials, including high specific area, enhanced chemical reactivity and high mechanical stability, which make it an excellent candidate for an immobilization substrate [21,22]. Moreover, further carbonization of the cellulose under proper conditions could yield unique nanostructured carbon materials with inherited fine morphologies [23].

**Figure 1:**
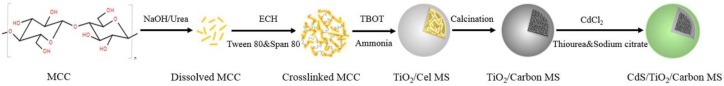
Schematic preparation route of the CdS/TiO_2_/Carbon MS.

To the best of our knowledge, the research dealing with the synthesis of core-shell structured hybrid materials based on a semiconductor and natural cellulose is scarce. So, in the present work, we developed a potential application of hybrid inorganic/organic CdS/TiO_2_/Carbon core-shell microspheres based on cellulose substrate as a photocatalysis system. The preparation route of the CdS/TiO_2_/Carbon microspheres (MS) is illustrated in Figure 1. Herein, the MCC is dissolved in the pre-cooled NaOH/urea aqueous solutions. However, the strength of the dissolved cellulose is poor because the dissolution of cellulose is limited by its molecular weights and concentrations [24]. So we employed a unique crosslinking method to reinforce the mechanical strength of dissolved cellulose. Then the TiO_2_/cellulose composite was prepared through the controlled hydrolysis of titanium tetrabutoxide (TBOT) in the presence of crosslinked microcrystalline cellulose. It is well known that cadmium sulfide (CdS) is a semiconductor with a band gap of 2.4 eV which could be excited by visible light to produce electrons and holes [25]. Therefore, a novel sonochemical method was designed to couple CdS with TiO_2_. Ultimately, the morphology, chemical structure and properties of the CdS/TiO_2_/Carbon MS were characterized in many ways. Additionally, the photocatalytic activity was evaluated with Rhodamine B (RhB) solution as a model contaminant.

## 2. Results and Discussion

### 2.1. Characterization of CdS/TiO_2_/Carbon MS

**Figure 2:**
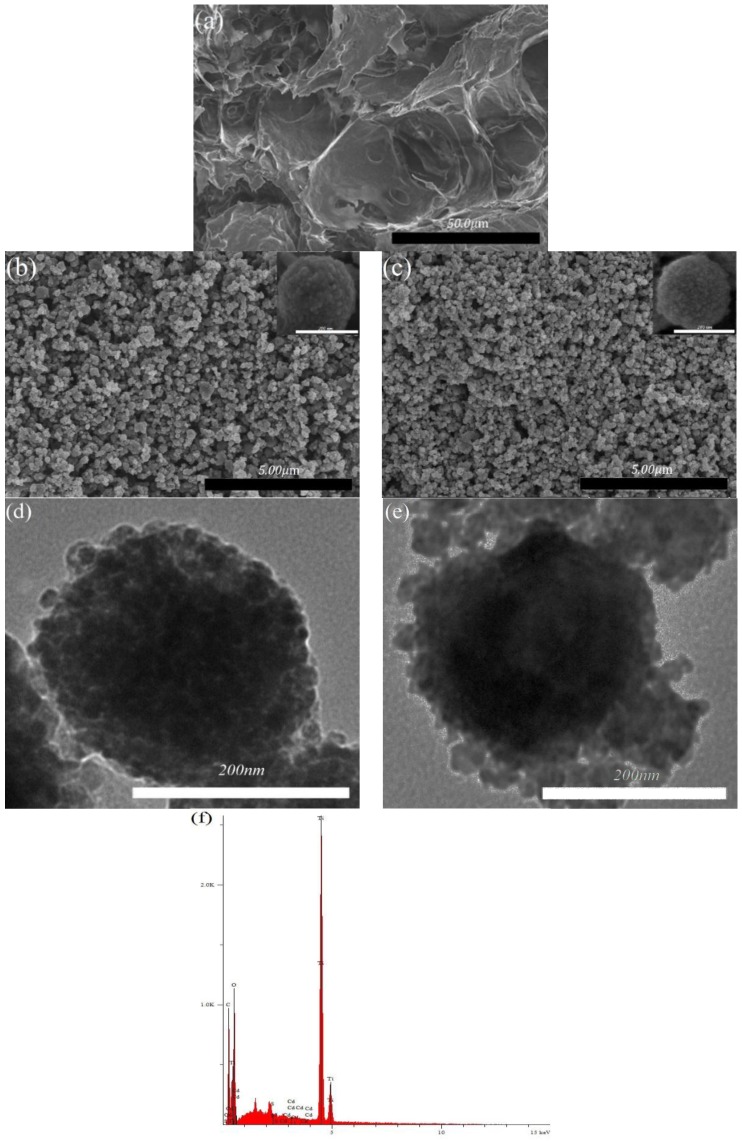
(**a**) SEM image of crosslinked MCC; (**b**) SEM image of TiO_2_/Carbon MS; (**c**) SEM image of CdS/TiO_2_/Carbon MS; (**d**) TEM image of TiO_2_/Carbon MS; (**e**) TEM image of CdS/TiO_2_/Carbon MS; and (**f**) EDX of CdS/TiO_2_/Carbon MS.

Figure 2a–e showed the scanning electron microscopy (SEM) and transmission electron microscopy (TEM) images of crosslinked MCC, TiO_2_/Carbon MS and CdS/TiO_2_/Carbon MS. As shown in Figure 2a, the crosslinked MCC is smooth and porous to some extent. In Figure 2b,d, the size of TiO_2_/Carbon MS was in the range of 100*–*500 nm. After coupling the CdS, the surface of the CdS/TiO_2_/Carbon MS became relatively smooth with an average diameter of 250 nm as shown in Figure 2c,e. Although the SEM and TEM results did not provide clear information about the composition in the samples, from the energy dispersive X-ray spectrum (EDX) analysis in Figure 2f, we could observe that the CdS/TiO_2_/Carbon MS contained the Ti, O, Cd and S elements, indicating the successful fabrication of the hybrid nanomaterial.

**Figure 3:**
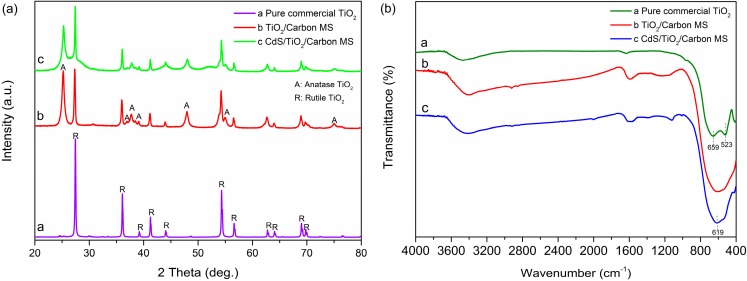
(**a**) XRD patterns and (**b**) FT-IR spectra of pure commercial TiO_2_, TiO_2_/Carbon MS and CdS/TiO_2_/Carbon MS.

Figure 3a showed the X-ray diffraction (XRD) patterns of TiO_2_/Carbon MS and CdS/TiO_2_/Carbon MS together with the pure commercial rutile TiO_2_. Comparing the TiO_2_/Carbon MS with pure commercial rutile TiO_2_, we could find that the main peaks located at 27.36°, 35.99°, 39.28°, 41.15°, 43.93°, 54.24°, 56.62°, 62.77°, 64.06°, 69.02° and 69.80° were assigned to the (110), (101), (200), (111), (210), (211), (220), (002), (310), (301) and (112) crystal planes of the rutile TiO_2_ (JCPDS No. 21-1276), respectively. In addition, distinct diffraction peaks observed at 25.21°, 36.91°, 37.73°, 38.44°, 48.08°, 55.04°and 75.01° in the TiO_2_/Carbon MS corresponded to the (101), (103), (004), (112), (200), (211) and (215) crystal planes of the anatase TiO_2_ (JCPDS No. 21-1272), respectively. Some studies proved that the highly photocatalytic active form is the mixed phase of two polymorphs, anatase and rutile, rather than their pristine compositions [26] The crystallite phase*–*dependent synergistic effect is often encountered in this phenomenon. The effect is believed to involve photo-excited charge migration between the two phases [27]. When the mixed-phase TiO_2_ is formed, the Fermi energies in the boundary between the anatase and rutile phases should be the same. This leads the potential of the conduction band (CB) and valence band (VB) of rutile to lie above those of anatase, which promotes excited electron transfer from anatase to rutile with a lower band energy [28,29]. In turn, the effect enhances charge separation and inhibits charge recombination. Meanwhile, it has been proved that the rutile phase can be excited by the visible light due to its small band gap and it acts as an antenna to extend the photo activity [30]. However, for CdS/TiO_2_/Carbon MS, the diffraction peaks of CdS were not distinct, which may be caused by the quite similar peak positions of CdS and TiO_2_ [31]. In the Fourier Transform infrared (FTIR) spectra of the samples (Figure 3b), the broad peaks in the region of 400–600 cm^−1^ for all samples were attributed to the stretching vibration of Ti–O–Ti and Ti–O bonds. Additionally, the absorption band at 619 cm^−1^ was assigned to the Cd–S stretching vibration [32].

**Figure 4:**
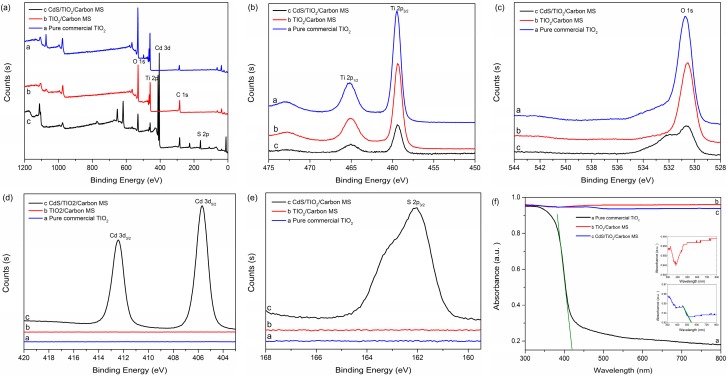
(**a**) Whole survey XPS spectrum; (**b**) Ti 2p XPS spectrum; (**c**) O 1s XPS spectrum; (**d**) Cd 3d XPS spectrum; (**e**) S 3p XPS spectrum and (**f**) UV-vis diffuse reflectance spectra of pure commercial TiO_2_, TiO_2_/Carbon MS and CdS/TiO_2_/Carbon MS.

The XPS survey scans of the surface of the pure commercial TiO_2_, TiO_2_/Carbon MS and CdS/TiO_2_/Carbon MS were performed to further investigate the elemental composition and surface chemical state of the samples. The whole XPS survey spectrum (Figure 4a) showed the presence of Ti and O in all samples, and Cd and S only existed in CdS/TiO_2_/Carbon MS (Figure 4d,e). In Figure 4b, two peaks at 459.4 eV and 465.1 eV were attributed to Ti 2p_3/2_ and Ti 2p_1/2_ of Ti^4+^ in TiO_2_ [33]. Additionally, the XPS peak of the O 1s could be observed at 530.6 eV in Figure 4c. The Cd 3d_5/2_ and Cd 3d_3/2_ peaks were identified at 405.7 eV and 412.4 eV from Figure 4d, respectively. In addition, it could be seen from Figure 4e that 162.1 eV was the binding energy for S 2p_3/2_. Both of these values were similar to previous reports for CdS, which suggested the existence of Cd^2+^ and S^2−^ of CdS in CdS/TiO_2_/Carbon MS [34]. Based on the results from EDX, XRD and FT-IR characterizations, it was confirmed that both TiO_2_ and CdS existed in the CdS/TiO_2_/Carbon MS.

The Ultraviolet-vis (UV-vis) diffuse reflectance absorption spectra were shown in Figure 4f to investigate the optical properties of pure commercial TiO_2_, TiO_2_/Carbon MS and CdS/TiO_2_/Carbon MS. The pure commercial TiO_2_ exhibited the characteristic spectrum with a fundamental absorption sharp edge at about 420 nm, which was in agreement with the results previously reported [35]. When TiO_2_ was deposited on the crosslinked MCC and calcined to form TiO_2_/Carbon MS, a continuous absorption band in the range of 400–800 nm appeared, which could be attributed to the fact that the physical appearance of the sample was black [36]. For CdS/TiO_2_/Carbon MS, the influence of color was weakened, and it was noteworthy that the ability of light absorption was enhanced to 542 nm.

**Figure 5:**
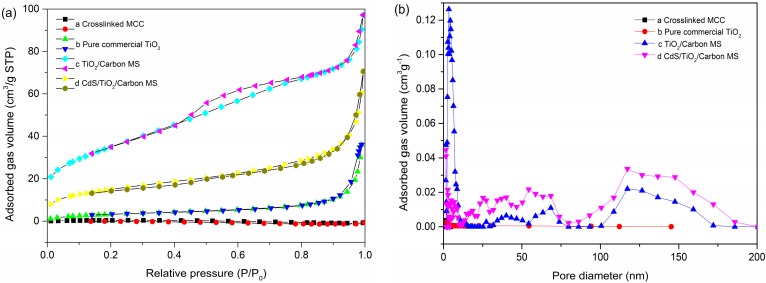
(**a**) Nitrogen adsorption and desorption isotherms and (**b**) Barrett-Joyner-Halandar (BJH) pore size distribution of dissolved MCC, pure commercial TiO_2_, TiO_2_/Carbon MS and CdS/TiO_2_/Carbon MS.

According to the International Union of Pure and Applied Chemistry (IUPAC) classification, the nitrogen adsorption–desorption isotherms of TiO_2_/Carbon MS and CdS/TiO_2_/Carbon MS were shown in Figure 5a, and they were identified as type IV with hysteresis loops, supporting a well-developed three-dimensional network structure of the material [37]. For CdS/TiO_2_/Carbon MS, an adsorption hysteresis loop was located at 0.94 < P/P_0_ < 0.99, corresponding to the existence of the macropores [38]. In addition, the overlapping of TiO_2_/Carbon MS and CdS/TiO_2_/Carbon MS nitrogen adsorption–desorption isotherms at low pressure referred to the existence of vast mesopores. Furthermore, it could be observed from Figure 5b that the Barrett-Joyner-Halandar (BJH) pore size distribution of both TiO_2_/Carbon MS and CdS/TiO_2_/Carbon MS exhibited similar pore size distributions ranging from 2 to 180 nm, which further proved the existence of numerous meso- and macropores. Obviously, there were a great many micropores in CdS/TiO_2_/Carbon MS. It has been demonstrated that such a porous structure has enhanced properties due to the efficient diffusion of reactant molecules and products [33].

Furthermore, the Brunauer–Emmett–Teller (BET) surface area, pore volume and average pore sizes of the samples were summarized in Table 1. As calculated by the BET method, CdS/TiO_2_/Carbon MS gave rise to a BET surface area of 54.8357 m^2^·g^−1^ and a relatively large pore volume of 0.106351 cm^3^·g^−1^. However, the average pore size of CdS/TiO_2_/Carbon MS was larger than that of the TiO_2_/Carbon MS; the BET surface area and pore volume of the CdS/TiO_2_/Carbon MS were smaller than that of the TiO_2_/Carbon MS. The reason was that the specific surface area and pore volume relate to per gram of sample [39]. When the sample contained a part of the CdS, the density of the CdS/TiO_2_/Carbon MS increased, so that the BET specific surface area and the pore volume of the sample were reduced.

### 2.2. Photocatalytic Activity of CdS/TiO_2_/Carbon MS

**Figure 6:**
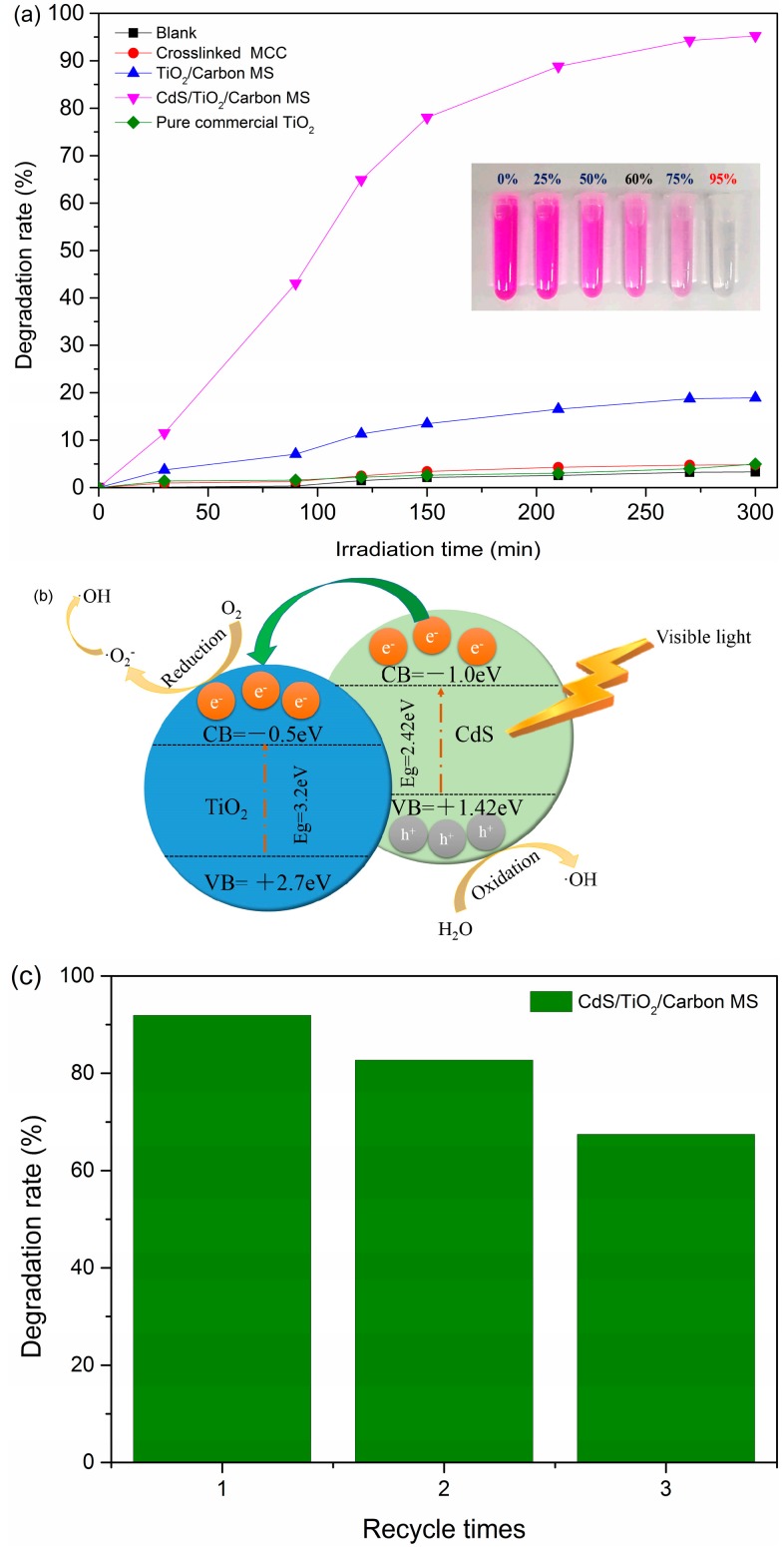
(**a**) The degradation performance of samples under visible irradiation within 300 min; (**b**) Proposed scheme of the charge transfer and separation processes of the CdS/TiO_2_ heterojunction; (**c**) Recycling test of the CdS/TiO_2_/Carbon MS.

The photocatalytic activity of the samples was evaluated by degrading the Rhodamine B (RhB) solution. Figure 6a exhibited the degradation results and the variation of RhB color with different degradation rates (inset). The degradation rates of blank and crosslinked MCC were poor (lower than 5%) after 300 min visible light irradiation. Meanwhile, the degradation of RhB by the crosslinked MCC was higher than that of the blank, which might ascribe to the adsorption to RhB on crosslinked MCC. Besides, only 4.96% of the RhB could be degraded in the presence of the pure commercial TiO_2_, because the band gap of TiO_2_ is high and it cannot be excited by visible light generally. However, the TiO_2_/Carbon MS degradation efficiency reached 18.92%. Namely, the TiO_2_/Carbon MS exhibited higher photocatalytic activity than that of pure commercial TiO_2_. The causes of this phenomenon might be attributed to two aspects: One was that the TiO_2_ in the TiO_2_/Carbon MS was composed of anatase and rutile TiO_2_. The other was that the core-shell structure of TiO_2_/Carbon MS possessed considerable potential to enhance the photocatalytic performance [40].

Particularly, the CdS/TiO_2_/Carbon MS exhibited a much higher photocatalytic performance, and the degradation efficiency reached about 95.24% within 300 min under visible light irradiation. Figure 6b showed a proposed scheme of the charge transfer and separation processes of the CdS/TiO_2_ heterojunction. Under visible light irradiation, the electrons were generated from the valence band (VB) of CdS to their conduction band (CB), then the electrons were transferred to the CB of TiO_2_. Simultaneously, the holes remained in the VB of CdS. Thus, the recombination between electrons and holes was inhibited and the CdS/TiO_2_/Carbon MS could induce large light-harvesting efficiency and supply more reaction active sites to improve the photocatalytic performance of the catalyst [41,42]. Furthermore, the unique porous structure and high specific area, which led to higher quantum efficiency of the photocatalytic reaction and the mixed phase of two TiO_2_ crystallites, were crucial for the significantly enhanced photocatalytic activity of the CdS/TiO_2_/Carbon MS [26,43,44].

For investigating the stability and recyclability of CdS/TiO_2_/Carbon MS, the recycling test of the CdS/TiO_2_/Carbon MS was performed. As shown in Figure 6c, the degradation rate of three instances of recycling reached up to 91.93%, 82.72% and 67.47%, respectively. Obviously, the CdS/TiO_2_/Carbon MS showed good stability and recyclability.

## 3. Materials and Methods

### 3.1. Materials

All chemicals, including microcrystalline cellulose (MCC), NaOH, urea, ethanol, epichlorohydrin (ECH), paraffin oils, Tween 80, Span 80, titanium tetrabutoxide (TBOT), sodium citrate, ammonia, thiourea, CdCl_2_, hydrochloric acid and Rhodamine B (RhB) were of analytical grade and used without further purification. Deionized water was used throughout the whole experiment.

### 3.2. Methods

#### 3.2.1. Fabrication of Crosslinked MCC

**Figure 7:**
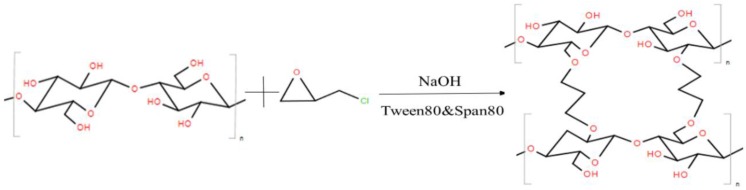
Proposed crosslinking reaction mechanism of dissolved MCC.

In this work, the MCC was dissolved in the precooled NaOH/urea aqueous solution (7 wt %/12 wt %) and degassed by centrifugation at 7200 rpm for 15 min. Then 20 mL of the MCC solution was dropped into a well-mixed suspension containing 100 mL of paraffin oils, 1.5 g of Tween 80 and 0.5 g of Span 80 within 5 min. The suspension was kept stirring for 2 h at 500 rpm and heated to 25 °C with the same stirring speed for 0.5 h. Subsequently, 2 mL of ECH was dropped into the suspension within 10 min, then the suspension was stirred for an additional 1.5 h to crosslink the MCC completely. The reaction mechanism of crosslinking MCC was shown in Figure 7. Diluted hydrochloric acid (10 wt %) was added to the resultant suspension until pH = 7. Next, washed it with deionized water and ethanol successively to remove the residual paraffin oils, Tween 80 and Span 80. Finally, the crosslinked MCC gel was freeze-dried.

#### 3.2.2. Preparation of TiO_2_/Carbon MS

First, 1.5 g of the as-prepared crosslinked MCC was ultrasonically dispersed in 100 mL of ethanol, and then the mixture solution was transferred to the beaker with 2.0 mL ammonia. Next, 10.0 g of TBOT was dissolved in 100 mL of ethanol and added to the former mixed solution, vigorously stirred at 60 °C for 3 h. Obtained solids were separated by centrifugation and washed with deionized water, then dried in an oven at 60 °C for 6 h. Finally, the sample was calcined under nitrogen atmosphere at 550 °C for an hour to yield TiO_2_/Carbon core-shell microsphere.

#### 3.2.3. Preparation of CdS/TiO_2_/Carbon MS

First, 0.3 g of the obtained TiO_2_/Carbon MS was ultrasonically dispersed in 150 mL of deionized water. Afterwards, 7.50 mL of 0.12 M CdCl_2_ solution, 10 mL of 0.1 M sodium citrate solution and 15 mL of 0.12 M thiourea solution were added to the solution under stirring gently for 5 min. Then 2 mL of ammonia was added into the mixed solution and sonicated continuously at 65 °C for 1.5 h. The suspension was separated by centrifugation and washed with deionized water, then dried in oven at 60 °C for 6 h. The expected molar ratio of CdS to TiO_2_ is 1.0:6.5.

#### 3.2.4. Characterization

The crystal structure of samples was analyzed by an X-ray diffractometer (Shimadzu XRD-6000, Shimadzu corporation, Kyoto, Japan) with Cu radiation (λ = 0.154 nm) at 40 kV and 40 mA. Scanning electron microscopy micrographs were taken with a Hitachi SU8010 scanning electron microscope (Hitachi High Tech CO., Tokyo, Japan) using an accelerating voltage of 5 kV, which was equipped with a Model 550i energy dispersive X-ray spectrometer (EDX, IXRF systems Inc., Austin, TX, USA). Transmission electron mocroscopy micrographs were recorded on a Hitachi HT7700 TEM System (Hitachi High Tech CO., Tokyo, Japan) operating at 100 kV. The X-ray photoelectron spectroscopy measurements were performed on Thermo Scientific Escalab 250Xi XPS system (Thermo Fisher Scientific Ltd, East Grinstead, UK) using Al Kα source. The Brunauer-Emmett-Teller (BET) and Barrett-Joyner-Halenda (BJH) measurements were performed by Micromeritics Tristar II 3020 analyzer (Micromeritics Instrument Corporation, Norcross, GA, USA). UV-Vis absorption spectra and diffuse reflectance spectroscopy were carried out with UV2310II spectrometer (Techcomp, Shanghai, China) and U-3900 Spectrophotometer (Hitachi High Tech CO., Tokyo, Japan), separately, and the spectra were recorded from 200 to 800 nm. Fourier-transform infrared spectroscopy data were acquired using a Perkin Elmer Spotlight 400 imaging stytem (Perkin Elmer, Beaconsfield, UK).

#### 3.2.5. Photocatalytic Activity

The photocatalytic activity of the CdS/TiO_2_/Carbon MS was evaluated by using the Rhodamine B (RhB) solution as the model pollutant under visible light irradiation. The photocatalytic reactor (Shanghai Bilon Instrument Co., Ltd., Shanghai, China) consisted of a quartz glass with a circulating water jack and a light source (A 1000 W xenon lamp). An aqueous solution of RhB (25 mL, 10 mg·L^−1^) was placed in a glass tube, and 4 mg of CdS/TiO_2_/Carbon MS were added. Prior to irradiation, the reaction mixture was stirred in the dark for 45 min to ensure the adsorption/desorption equilibrium between the sample and RhB solution. At certain time intervals, 4 mL of solution was centrifuged and analyzed by recording variations of the maximum absorption spectra (554 nm) by UV2310II spectrometer. For the photocatalysis stability measurements, 4 mg CdS/TiO_2_/Carbon MS was dispersed in 25 mL RhB solution (10 mg·L^−1^) and the photocatalytic activity tested as described obove with the photoreaction time is certain for 240 min. After each photocatalysis test, the CdS/TiO_2_/Carbon MS was separated by centrifugation and washed with deionized water several times. Then the obtained CdS/TiO_2_/Carbon MS returned to the reaction system and the recycling test was performed under the same condition. The degradation rate (*D*) of RhB was calculated by the following formula:

$$D=\left(1-C/C_{0}\right)\times 100\%=\left(1-A/A_{0}\right)\times 100\%$$

where *D* (%) is the degradation rate, *C*_0_ is the initial RhB solution concentration, *C* is the concentration of the RhB solution at reaction time *t*. *A* and *A*_0_ are the responding absorption values (ABS). In addition, the photocatalytic activities of crosslinked MCC, pure commercial TiO_2_, TiO_2_/Carbon MS as well as the blank were also tested under the same condition.

## 4. Conclusions

In summary, we have successfully synthesized the CdS/TiO_2_/Carbon MS by controlled hydrolysis and a novel sonochemical method. Many attempts have been made to combine the advantageous characteristics of microcrystalline cellulose with the specific properties of semiconductors. The as-prepared CdS/TiO_2_/Carbon MS as a hybrid inorganic/organic composite displayed good light absorption ability, superior surface properties and favorable photo-generated charge-separation efficiency, which might have promising application in the cleanup of the environment. The approach we developed could be applied to fabricate a series of carbon composites derived from natural cellulose with tailored structures, properties and functionalities for specific practical applications and open endless possibilities for the creation of new catalysis systems with a whole range of functional properties.

## Figures and Tables

**Table 1:** Calculation results of BET surface area, pore volume and average pore size of samples.

Sample	Surface Area (m^2^·g^−1^)	Pore Volume (cm^3^·g^−1^)	Pore Size (nm)
Crosslinked MCC	1.5260	0.000219	2.07380
Pure commercial TiO_2_	12.5280	0.056261	9.58286
TiO_2_/Carbon MS	127.1291	0.148790	3.95267
CdS/TiO_2_/Carbon MS	54.8357	0.106351	5.27906
